# The Tax protein and the minichromosome maintenance protein complex MCM2-7 affect cell replication and viral transcription

**DOI:** 10.1186/1742-4690-11-S1-P96

**Published:** 2014-01-07

**Authors:** Pierre-Yves Barez, Alexandre Carpentier, Mathieu Boxus, Luc Willems

**Affiliations:** 1 Molecular and cellular Epigenetics, Interdisciplinary Cluster for Applied Genoproteomics (GIGA), University of Liège (ULg), Liège, Belgium; 2 Molecular Biology Lab, Gembloux Agro Bio-Tech (ULg), Gembloux, Belgium

## 

The Tax oncoprotein plays a key role in the mechanisms of transformation, viral persistence and pathogenicity. Recently, we showed that Tax interacts with the minichromosome maintenance MCM2-7 helicase and binds to origins of DNA replication (Boxus et al, 2012 Blood 119:151). In fact, Tax modulates the spatiotemporal program of origin activation during the S phase of cell cycle. By this mechanism, Tax accelerates S phase progression through early firing of late replication origins. By interacting with the 5’ LTR, the MCM2-7 complex also modulates Tax transactivation. Together, our data thus demonstrates that interaction between Tax and MCM2-7 modulates reprogramming of replication origins as well as viral transcription.

